# Analysis of image-guided superficial radiation therapy (IGSRT) on the treatment of early-stage non-melanoma skin cancer (NMSC) in the outpatient dermatology setting

**DOI:** 10.1007/s00432-023-04597-2

**Published:** 2023-02-01

**Authors:** Alison Tran, Mairead Moloney, Peter Kaczmarski, Songzhu Zheng, Alpesh Desai, Tejas Desai, Lio Yu

**Affiliations:** 1 Menter Dermatology Research Institute, Baylor University Medical Center, Dallas, TX USA; 2 Heights Dermatology, Houston, TX USA; 3 New York Institute of Technology College of Osteopathic Medicine, Old Westbury, NY USA; 4 Stony Brook University, Stony Brook, NY USA; 5 Laserderm Dermatology, Smithtown, NY USA

**Keywords:** Image-guided superficial radiation therapy, Early-stage non-melanoma skin cancer, Clinical dermatology, Dermatologic surgery, Oncologic therapy, Ethnicity/race disparities

## Abstract

**Background:**

Interest in image-guidance superficial radiation therapy (IGSRT) for the treatment of early-stage non-melanoma skin cancer (NMSC) has resurfaced given its low complication rates, superior cosmesis and local control and cure rates. In addition, it has been recommended by the American Academy of Dermatology (AAD) for early-stage NMSC in patients who are considered poor surgical candidates.

**Methods:**

1899 NMSC lesions were treated with energies ranging from 50 to 100 kilovoltage (kV), for a mean of 20.2 fractions, and treatment dose of 5364.4 centigray (cGy). Lesions were treated for a mean of 7.5 weeks and followed for 65.5 weeks. SAS studio was used to conduct Kaplan–Meier analysis to calculate local control rates and account for differences in follow-up intervals. A log-rank test was used to calculate statistical differences between histologies.

**Results:**

Absolute lesion control was achieved in 99.7% of the patients after an average of 7.5 weeks of treatment, with a stable control rate of 99.6% when the follow-up duration was over 12 months. 95% of lesions with toxicity scoring received a Radiation Treatment Oncology Group Toxicity (RTOG) score of 1 or 2.

**Conclusion:**

IGSRT has a high safety profile, can achieve superior cosmesis and should be considered first-line for treating early-stage NMSC tumors as cure rates have been shown to be effective in all NMSC on early follow-up.

## Introduction

Skin cancer is the most common form of cancer in the United States, with more than 9,500 people diagnosed daily (Rogers et al. 2015). The incidence of both melanoma and non-melanoma skin cancer (NMSC) has been steadily rising. Between 1994 and 2014, the diagnosis and treatment of NMSC in the United States increased by 77% (Mohan and Chang 2014). NMSC is the most frequently diagnosed cancer, with an 18–20 times higher incidence than melanoma (Didona et al. 2018; Yu et al. 2021a; The Lewin Group 2005). The most recent estimate in 2012 revealed more than 5.4 million cases of NMSC treated in over 3.3 million patients (Yu et al. 2021a). The cost of skin cancer treatment in the United States is estimated at $8.1 billion annually (Gupta et al. 2016), of which approximately 4.3 million patients are treated for basal cell carcinoma (BCC) and squamous cell carcinoma (SCC) at $4.8 billion, while melanoma treatment costs $3.3 billion (Didona et al. 2018; Gupta et al. 2016). However, these estimates do not consider those who have no access to treatment or are uninsured.

Skin cancers may arise from any host cell of the skin. However, BCC and SCC are keratinocyte carcinomas that account for 99% of NMSC (Didona et al. 2018; Yu et al. 2021a). Overall, BCC is the most common form of skin cancer, followed by SCC (Rogers et al. 2015). However, SCC is the most common skin malignancy among African Americans and Asian Indians (Gupta et al. 2016; Centers for Disease Control and Prevention 2022). BCC contributes to 65%-75% of skin cancers in whites and 20–30% in people of color (Gupta et al. 2016). An estimated 5.03 to 5.23 million BCC lesions and between 200,000 and 400,000 SCC lesions are diagnosed yearly in the United States (Yu et al. 2021a). Central cancer registries do not generally collect data on basal cell and squamous cell carcinomas (Centers for Disease Control and Prevention 2022). As such, the reported figures may be an underestimate.

The overall prognosis for both BCC and SCC is good, especially when detected at early stages (Didona et al. 2018). BCC minimally contributes to the NMSC mortality rate (MR) at 0.02 per 10,000 (Didona et al. 2018; Leiter et al. 2014; Apalla et al. 2017). Meanwhile, SCC shows a variable metastatic rate of 0.1–9.9%, accounting for about 75% of NMSC-related deaths (Didona et al. 2018; Leiter et al. 2014; Apalla et al. 2017). Despite the high occurrence of NMSC, they are considered nonfatal and curable due to their slow growth, low recurrence, and rare metastasis (Iriyama et al. 2011; Lavker et al. 1989). Nonetheless, NMSC should be treated to prevent growth, invasion, and potential mortality. The latest data suggests that greater than 15,000 people die of SCC in the U.S. yearly, which is twice that of melanoma-related deaths (Mansouri and Housewright 2017) with more than 5,400 people worldwide dying of NMSC every month (Global Burden of Disease Cancer Collaboration 2019). This translates to about 65,000 NMSC-related deaths worldwide annually.

Major risk factors for the development of NMSC include increased exposure to ultraviolet (UV) radiation (hence the greatest incidence in sun-exposed areas, i.e., head and neck), as approximately 90% of NMSC are associated with UV exposure (Rogers et al. 2015). Other risk factors include older age, fair skin/hereditary risk factors, and improved surveillance, which contribute to earlier recognition (Nikolaou and Stratigos 2014). In addition, genetic polymorphisms also modulate susceptibility to skin cancer (Gupta et al. 2016; Meyer 2009). Chronic scarring and areas of chronic inflammation may also pose a risk factor for developing SCC in darkly pigmented individuals (Gupta et al. 2016). Moreover, SCCs have a greater tendency to occur in non-exposed sites with a higher potential for metastasis in Asians (Gupta et al. 2016; Kim et al. 2009).

Ethnic minorities, elderly, the less educated, uninsured and those of low socioeconomic status have poorer melanoma and NMSC outcomes (Buster et al. 2012). Advanced stage at presentation, atypical distribution of malignant skin lesions and socioeconomic factors, e.g., lack of adequate insurance coverage and/or transportation prevent timely diagnosis and early treatment (Bradford 2009). Though quality of care has reportedly improved throughout the years, access to care and health disparities have not (Buster et al. 2012). Inadequate access to dermatologic care may be explained by the current dermatology workforce shortage coupled with the increased patient load (Buster et al. 2012). Further, most counties with African, Hispanic and Native American majorities have no dermatologists (Vaidya et al. 2018; Tran and Gohara 2021).

## Treatment modalities

Early-stage NMSC treatments include photodynamic therapy (PDT), cryotherapy, topical medications such as imiquimod 5% and diclofenac sodium 3% (Yu et al. 2021a), laser, electrodessication and curettage, and radiotherapies such as superficial radiation therapy (SRT), image-guided superficial radiation therapy (IGSRT), external beam radiation therapy (XRT) which include electron-beam radiation and isotope-based and electronic brachytherapy. Since the most frequently affected areas include the head and neck, it is essential to employ treatments that have high cure rates and simultaneously engender superior esthetic results. While the standard of care is surgical excision, preferentially Mohs Micrographic Surgery (MMS), due to high cure rates and ability to achieve tissue conservation, not all patients are surgical candidates. In these situations, it is appealing to choose a non-surgical modality that has superior esthetic results and high cure rates.

### Superficial radiation therapy (SRT)

Superficial radiation therapy (SRT) has been used for decades to treat NMSC. It is a form of external radiotherapy that uses low energy, low penetration, kilovoltage (kV) photons between 50 and 150 kilovoltage peak (kVp) (Hamouzadeh et al. 2017), which preferentially targets tumors of the skin while sparing deeper structures (Hamouzadeh et al. 2017; Council on Science and Research 2021) beyond the dermis. Recent advancements in radiation technology, i.e., better tumor depth coverage and use of image guidance, have improved local control and cure rates. Its low complication rates, e.g., no pain or scarring combined with superior cosmesis, make it an attractive alternative to surgical intervention. Hence, it has been recommended by the American Academy of Dermatology (AAD) (Council on Science and Research 2021) and studies published in the Journal of the American Academy of Dermatology (JAAD) (Cognetta et al. 2012) for the treatment of NMSC in patients who are poor surgical candidates and in other studies as a primary option in appropriate patients (Yu et al. 2021b; Nestor et al. 2019).

### Image-guided superficial radiation therapy (IGSRT)

IGSRT combines ultrasound technology with SRT delivery. The high-resolution ultrasound (HRUS) is designed to detect dermatologic structures using frequencies of 22 MHz, which enable visualization of skin structures, lesions and depth as well as the lateral configuration of the tumor. The tumor depth is used to correlate with the percentage depth dose (PDD), which determines the selection of energy (50, 70, or 100 kV) delivered, and adjustments can be made during the treatment. The most recent and largest modern study (2917 cases of early-stage SCCIS, T1 and T2 NMSC) conducted by Yu and colleagues in 2021 demonstrated that IGSRT had an absolute local control rate of 99.3%, which was stably unchanged at the follow-up intervals of greater than 1 year to a max of 4 years (Yu et al. 2021b).

## Methods

A retrospective chart review of 1243 patients with 1899 lesions from an outpatient dermatology practice in Dallas, Texas, was analyzed from 2016 to 2022 after the study protocol was reviewed and determined to be exempt from IRB approval by an IRB committee (WIRB-Copernicus Group) under 45 CFR 46.104 (d)(4). The information obtained was recorded by the investigator in such a manner that the identity of the human subject could not be readily ascertained directly or through identifiers linked to the subjects, the investigator does not contact the subjects, and the investigator will not re-identify subjects. Any health information used in this study has been de-identified. This study was performed in compliance with the pertinent sections of the Helsinki Declaration and its amendments. All methods were carried out in accordance with relevant guidelines and regulations.

### Inclusion criteria

Patients with varying Fitzpatrick skin types and NMSC, i.e., BCC, SCC and SCCIS who received 20 or more treatments were included in this study. Lesions that were not considered were keloids, non-keratinocytic tumors, and tumors of stage III or greater.

### Treatment

Board-certified radiation therapists administered IGSRT technology to treat lesions with energies ranging from 50, 70 or 100 kilovoltage (kV), which was delivered 2–4 times weekly. The mean total number of fractions was 20.2 (SD ± 0.90), ranging from 20 to 30. The mean total treatment dose was 5364.4 centigray (cGy) (SD ± 241.60), ranging from 4453.4 to 6703.2 cGy. The majority of these lesions were treated for 7.5 weeks and followed for a mean of 65.5 weeks (SD ± 66.70) (Table 1). The duration of follow-up was calculated as the date of last follow-up minus the last treatment date plus 1 day.

**Table 1:** Total number of treatment fractions, dose, duration and follow-up interval

Characteristic	Statistic	(*N* = 1899)
Total number of fractions	*N*	1899
	Mean ± SD	20.2 ± 0.90
	Range	20.0 to 30.0
	Median	20.0
Total treatment dose (cGy)	*N*	1899
	Mean ± SD	5364.4 ± 241.60
	Range	4453.4 to 6703.2
	Median	5392.8
Duration of treatment (weeks)	*N*	1899
	Mean ± SD	7.5 ± 1.99
	Range	0.0–27.3
	Median	7.29
Duration of follow-up (weeks)	*N*	1899
	Mean ± SD	65.5 ± 66.70
	Range	0.14–276.29
	Median	42.29
Total number of lesions	*N*	1899

A high-resolution ultrasound (HRUS) simulation was performed to establish the field and determine the lesion for proper selection prior to treatment. This visualization is necessary to determine tumor breadth and depth for width, energy and dose selection (Yu et al. 2022). Thereafter, HRUS was performed during each treatment and after completion in order to make real-time modifications and assess for treatment response, respectively. Energy selection and dose adjustments were contingent upon tumor characteristics seen clinically and on ultrasound (histology and depth). One hundred and seventy-six lesions (9.9% [176/1779]) were treated with a combination of 2 or more energies. Lesion treatment by energy is summarized in the following (Table 2). The protocol used to determine energy administered has evolved over the years, in which a more detailed protocol that specified time dose fractionation (TDF) number/dose/fractionation, was developed in 2019 based on ultrasound depth and tumor type. This protocol recommends a fractionation dose range of 245–279 cGy for 20 fractions 3–4 times a week to achieve a therapeutic biological dose range of 90–99 or greater TDF number using 50, 70, or 100 kV energy. Higher doses per fraction and/or more fractions were recommended for larger, deeper, and high-risk lesions (Yu et al. 2021b). Nonetheless, energy administered is contingent upon anatomic location, histology, lesion depth, and skin curvature. The Radiation Treatment Oncology Group (RTOG) toxicity scoring system was used to grade acute toxicities after every 5 fractions and the highest RTOG score was recorded. These on treatment evaluations occurred throughout the treatment course. Follow-up occurred at 2–12 week intervals with a mean follow-up of 65.5 weeks after treatment completion until no evidence of disease (NED) was achieved or failure/recurrence. Safety checks on the SRT machine were performed by the medical physicist regularly.

**Table 2:** NMSC lesion treatment by energy

Energy (kV)	BCC only	SCC only	SCCIS only	Combined BCC and SCC	Combined BCC and SCCIS	Number of lesions^a^ (*N* = 1779)
50	200	70	79	0	0	349
70	456	236	257	0	1	950
100	175	73	55	1	0	304
Mixed	94	50	32	0	0	176

^a^116 lesions with no energy data and 4 lesions with no histology data

### Statistical analysis

Missing data were excluded from final analysis. SAS studio was used to conduct Kaplan–Meier analysis to calculate local control rates and account for differences in follow-up intervals between patients. A log-rank test was used to calculate statistical differences between histologies with a *p* value of < 0.05 considered statistically significant.

## Results

In this current study, 1899 NMSC lesions in 1243 patients were treated with IGSRT from 2016 to 2022. The cohort consisted of 981 BCC, 467 SCC, and 444 SCCIS lesions (certain lesions had combinations of 2 or more of these histologies with five lesions of unspecified histology). Among 1243 patients, 99.7% (1239/1243) were alive as of May 2022. All deaths were deemed unrelated to the treatment of NMSC by IGSRT.

A majority (58.7%) of the patients identified as White, unspecified (40.9%), followed by American Indian/Alaska Native and Asian (0.2% and 0.1%, respectively). The majority of patients identified as Non-Hispanic or non-Latino consisting 54.6% of the study population, with 44.3% unspecified, while 1.0% of patients identified as Hispanic or Latino. In addition, male patients contributed to 62.6% of the sample, while female patients comprised 36.7% with 0.7% unspecified. Lastly, the mean age of the sample was 73.2 years (SD ± 10.99 years), with 33.1 years being the youngest and 98.2 years being the oldest (Table 3).

**Table 3:** Demographic distribution of patients

**Race**	
White	730/1243 (58.7%)
American Indian or Alaska Native	2/1243 (0.2%)
Asian	1/1243 (0.1%)
Other	1/1243 (0.1%)
Unspecified	509/1243 (40.9%)
**Ethnicity**	
Not Hispanic nor Latino	679/1243 (54.6%)
Hispanic or Latino	13/1243 (1.1%)
Unspecified	551/1243 (44.3%)
**Sex**	
Male	778/1243 (62.6%)
Female	456/1243 (36.7%)
Unspecified	9/1243 (0.7%)
**Age at first treatment (years)**	
Mean ± SD	73.2 ± 10.99
Range	33.1–98.2
Median	74.1

Table 4 shows the most common sites of the treated lesions were head and neck as a group, extremities, followed by the head and neck subgroups cheek and nose. Table 5 demonstrates that BCC (51.7%) was more common than SCC (24.6%) and SCCIS (23.4%). The mean diameter of measured lesions was 1.3 cm (SD ± 0.69), ranging from 0.0 to 3.9 cm. The mean diameter was 1.3 mm (SD ± 0.70) for BCC, 1.4 mm (SD ± 0.66) for SCC, and 1.3 mm (SD ± 0.68) for SCCIS. Of the 981 BCC lesions, the measured diameter ranging from 0 to < 2 cm (T1) was detected in approximately 78.1% of lesions and 19.3% of lesions were observed to have diameters ranging from 2 to < 4 cm (T2). Of the 467 SCC lesions, the measured diameter ranging from 0 to < 2 cm was observed in 73.2% of lesions, and diameters ranging from 2 to < 4 cm were seen in 24.4% of lesions. 76.1% of the 444 total SCCIS lesions (Tis) were observed to have a diameter ranging from 0 to < 2 cm and 20.5% of lesions were found to have diameters ranging from 2 to < 4 cm. SCCIS lesions greater than or equal to 4 cm were still considered Tis by AJCC staging manual 8th edition (Amin et al. 2017) (Table 6).

**Table 4:** Anatomic distribution of NMSC lesions

Head and neck (H&N)	1221/1899 (64.3%)
**H&N sublocation**	
Ear	168/1899 (8.8%)
Cheek	278/1899 (14.6%)
Nose	307/1899 (16.2%)
Cutaneous lip	34/1899 (1.8%)
Mucosal lip	14/1899 (0.7%)
Forehead	168/1899 (8.8%)
Forehead/scalp	2/1899 (0.1%)
Forehead/temple	2/1899 (0.1%)
Temple	63/1899 (3.3%)
Cheek/temple	1/1899 (0.05%)
Scalp	107/1899 (5.6%)
Neck	77/1899 (4.1%)
Extremities	448/1899 (23.6%)
**Extremities sublocation**	
Hand	92/1899 (4.8%)
Trunk	124/1899 (6.5%)
**Trunk sublocation**	
Chest	51/1899 (2.7%)
Back	64/1899 (3.4%)
Unspecified trunk	9/1899 (0.5%)
Shoulder	39/1899 (2.1%)

67 lesions with no location recorded

**Table 5:** Cancer types and initial lesion size at initiation of treatment

Cancer types	*N* = 1899
BCC	981/1899 (51.7%)
SCC	467/1899 (24.6%)
SCCIS	444/1899 (23.4%)
Mixed BCC and SCC	2/1899 (0.11%)
Mixed BCC and SCCIS	1/1899 (0.05%)
Unspecified	4/1899 (0.21%)
**Lesion diameter (cm) at start**	***N* = 1899**
Mean ± SD	1.3 ± 0.69
Range	0.0–3.9
Median	1.0
**BCC lesion diameter (cm) at start**	***n* = 981**
Mean ± SD	1.3 ± 0.70
Range	0.3–3.5
Median	1.0
**SCC lesion diameter (cm) at start**	***n* = 467**
Mean ± SD	1.4 ± 0.66
Range	0.3–3.5
Median	1.3
**SCCIS lesion diameter (cm) at start**	***n* = 444**
Mean ± SD	1.3 ± 0.68
Range	0.0–3.9
Median	1.0

**Table 6:** Diameter of NMSC lesions

Diameter	BCC only	SCC only	SCCIS only	BCC & SCC	BCC & SCCIS	Total
0 to < 2 cm	766	342	338	1	1	1448
2 to < 4 cm	189	114	91	1	0	395
Unspecified	26	11	15	0	0	52
Total	981	467	444	2	1	1895^a^

^a^4 lesions had unspecified histology

### Local control (LC)

**Fig. 1:**
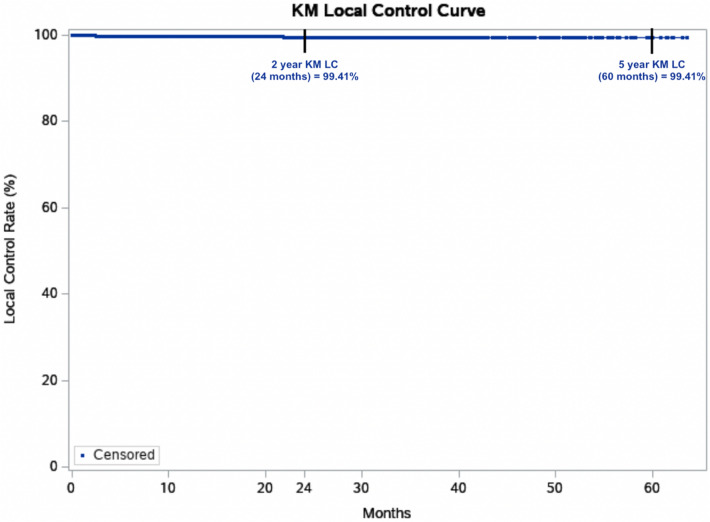
2-year and 5-year Kaplan–Meier (KM) local control (LC) for all 1899 lesions treated with image-guided superficial radiation therapy from 2016 to 2022. Dots represent censored events

**Fig. 2:**
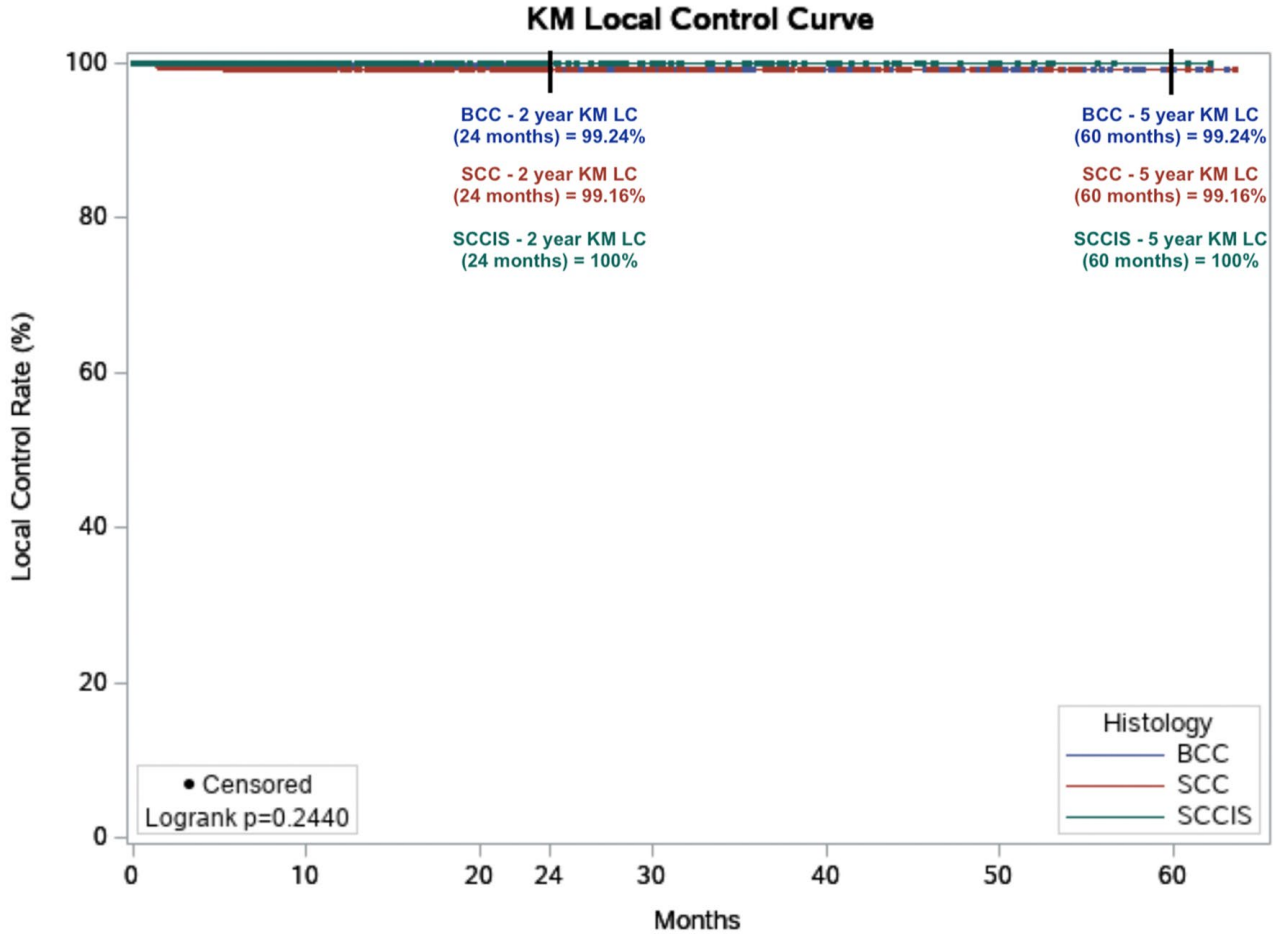
Kaplan–Meier (KM) local control (LC) by histology in all 1899 lesions treated with image-guided superficial radiation therapy from 2016 to 2022

Absolute LC was achieved in 99.7% of all lesions after an average of 7.5 weeks of treatment. In the cohort of lesions with > 12 months follow-up, a stable control rate of 99.6% was achieved (Table 7). Six lesions recurred at a mean of 6.83 months post-treatment. Overall, Kaplan–Meier (KM) LC was 99.41% at the maximum follow-up time of 63.6 months (5 years) (Fig. 1). When comparing lesions by histology at maximum follow-up (Fig. 2), KM LC for BCC and SCC were comparable at 99.24% and 99.16%, respectively. KM LC for SCCIS was 100% at maximum follow-up of 62.1 months. Log-rank comparison of KM LC between histologic subtypes (BCC, SCC, SCCIS) was not statistically significant (*p* = 0.2440, alpha = 0.05).

**Table 7:** Control rates by tumor type and length of follow-up

	All	All > 12 months	SCCIS	SCCIS > 12 months	Combined BCC + SCC	Combined BCC + SCC > 12 months
No. of lesions	1899	807	444	202	1450	604
Mean lesion diameter (cm)	1.3 ± 0.69	1.3 ± 0.67	1.3 ± 0.68	1.3 ± 0.63	1.3 ± 0.69	1.3 ± 0.68
Median lesion diameter (cm)	1.0	1.0	1.0	1.0	1.0	1.1
Mean no. of treatments	20.2	20.2	20.2	20.2	20.3	20.2
Median no. of treatments	20.0	20.0	20.0	20.0	20.0	20.0
Mean total treatment dose	5364.4	5297.5	5384.0	5344.9	5359.0	5282.0
Median total treatment dose	5392.8	5327.0	5395.6	5395.6	5385.6	5294.8
Mean follow-up (weeks)	64.0	126.0	64.4	117.3	64.0	128.9
Median follow-up (weeks)	41.0	111.3	47.6	95.6	38.9	117.4
Absolute local control (%)	99.7%	99.6%	100.0%	100.0%	99.6%	99.5%

### Response

Among 1196 lesions with RTOG toxicity grade data available, 61 lesions (5.1%) were RTOG grade 3 or 4 in severity (Table 8). 94.9% of lesions received RTOG grade of 1 or 2, consisting of mild or moderate self-resolving symptoms. The most common side effects displayed in patients were erythema, dryness followed by dry desquamation; however, some patients experienced ulceration and moist desquamation which did not affect lesion control. A median of 20 fractions was delivered at a mean total treatment dose of 5364.4 cGy with a mean follow-up of 65.5 weeks.

**Table 8:** Safety—by lesion based on RTOG criteria

Characteristic	Grade	Description	(*n* = 1196)^a^
Highest RTOG toxicity grade	1	Follicular, faint, or dull erythema; epilation; dry desquamation; decreased sweating	843/1196 (70.5%)
	2	Tender or bright erythema, patchy moist desquamation; moderate edema	292/1196 (24.4%)
	3	Confluent, moist desquamation other than skin folds; pitting edema	50/1196 (4.2%)
	4	Ulceration, hemorrhage, necrosis	11/1196 (0.9%)

*RTOG* radiation treatment oncology group

^a^703 lesions missing RTOG grading

## Discussion

The 2-year and 5-year absolute LC observed in this study are identical, which is consistent with the generally accepted premise that most failures in NMSC occurs within 2–3 years (Yu et al. 2022; Samarasinghe and Madan 2012). There is low likelihood of local recurrence for early-stage NMSC after 2 years (Amin et al. 2017; Samarasinghe and Madan 2012). Recurrence of BCC primarily occurs within the first 4–12 months (Samarasinghe and Madan 2012). This study reinforces that the dosing regimen employed, along with the use of high-resolution ultrasound guidance, allow for achieving outstanding local control.

The most common and current curative radiation regimen in the hospital and cancer center setting consists of 180–200 cGy administered daily five times per week for 30–36 treatments to total cumulative doses of 5400–7200 cGy for all tumors (Yu et al. 2021b). For the skin, small fields may be treated with a hypofractionated regimen of 2–4 times weekly at higher fractions of 220–400 cGy (Yu et al. 2021b). In the aforementioned study, the protocol initially consisted of 255 cGy for 20 total fractions, with 50 kV given for lesion depth less than 1.5 mm and 70 kV for lesion depth greater than 1.5 mm three times a week (Yu et al. 2021b). In addition, these results compare favorably with MMS. Our study affirms its efficacy and safety as our results showed a 99.7% control rate, which is comparable to the 99.3% control rate found in the multi-institutional SRT study led by Yu et al. (2021b) and patients experienced similar self-limiting side effects with a majority of patients receiving a RTOG score of 1.

The acute toxicity in this study showing < 1% Grade 4 RTOG toxicity is also compatible with results reported by Yu et al. (2021b). The approximately 4% Grade 3 RTOG toxicity, however, while still low, may imply there may be slightly more toxicity, but is still very much within safe parameters with the use of a more modern 2019 protocol detailed above. A possible explanation for the increase in Grade 3 RTOG toxicity over that reported by Yu et al. (2021b) is the slightly increased median dose from 5188 to 5393 cGy, which also seems to imply an increase in local control from 99.3 to 99.7%. Although this improvement is < 1%, the sheer numbers of annual lesions with NMSC in the United States alone (approximately 5.3 million causes) theoretically translates to ~ 50,000 more lesions controlled. The lesions of patients treated from 2019 onward using the updated protocol in the original 2021 Yu study was 30% (876/2917) (internal data courtesy of Dr. Yu). Whereas in this study, roughly 74% (1398/1899) of the lesions were treated after 2019. IGSRT has a high safety profile with limited, self-resolving side effects such as minimal pain, swelling, desquamation, and radiation dermatitis, making it a safe, and potentially cost-effective option for NMSC treatment compared to surgery. Surgical wound healing associated costs include medications for pain management, wound care and dressings, debridement and potential reconstruction with the use of costly grafts, e.g., placental grafts (Tran et al. 2023). Further, treatment with IGSRT typically includes more than one lesion (range of 1–4 lesions at a time or average 1.7 lesions) and is not anatomically restricted. Given the overall trend towards less/non-invasive and non-surgical options in medicine, IGSRT remains a promising alternative to MMS and other surgical options and should be considered more readily for patients with early-stage NMSC, advanced age, and patients with medical comorbidities, e.g., diabetes, stasis dermatitis, chronic edema that may render them poor surgical candidates/have poor wound healing capability. In addition, there are implications for improved care through improved quality of life via prevention of disfigurement (as well as associated pain and pruritus), especially in scar/keloid individuals, e.g., darker pigmented individuals with substantial evidence for its effectiveness in treating recurrent/resistant keloid scars (Nestor et al. 2019). It also has the potential to address disparities to access to dermatologic care as the patient may receive IGSRT from other qualified personnel, e.g., Board-Certified radiation therapists and IGSRT is often covered by insurance. This option may be especially beneficial in underserved areas, i.e., rural areas or areas underserved by dermatologists. In addition, this non-invasive treatment option may be advantageous for those with scar/keloid-prone skin (Nestor et al. 2019), such as those with darker pigmented skin, e.g., African Americans, Asians, and Hispanics. Currently, high-precision radiation oncology aims to optimize tumor coverage without sacrificing normal tissues. IGSRT with ultrasound assistance is one example of personalized oncology whereby precise high-dose delivery can be achieved at the desired superficial level.

## Conclusion

IGSRT should be considered first-line for treating early-stage NMSC tumors as cure rates have been shown to be effective in all NMSC on early follow-up and has the potential to be superior (with more follow-up data) to traditional SRT and surgery (Yu et al. 2021b). IGSRT may be useful in improving disparities in underserved communities, persons of color, those of low socioeconomic status and underprivileged segments of the population.

## Data Availability

All data generated or analyzed during this study are included in this published article (and its supplementary information files).

## References

[CR1] Amin MB, Edge S, Greene F, Byrd DR, Brookland RK, Washington MK, Gershenwald JE, Compton CC, Hess KR (2017). AJCC cancer staging manual.

[CR2] Apalla Z, Nashan D, Weller RB, Castellsagué X (2017). Skin cancer: epidemiology, disease burden, pathophysiology, diagnosis, and therapeutic approaches. Dermatol Ther.

[CR3] Bradford PT (2009). Skin cancer in skin of color. Dermatol Nurs.

[CR4] Buster KJ, Stevens EI, Elmets CA (2012). Dermatologic health disparities. Dermatol Clin.

[CR5] Centers for Disease Control and Prevention (2022) Skin cancer statistics. https://www.cdc.gov/cancer/skin/statistics/index.htm. Accessed 19 July 2022

[CR6] Cognetta AB, Howard BM, Heaton HP, Stoddard ER, Hong HG, Green WH (2012). Superficial X-ray in the treatment of basal and squamous cell carcinomas: a viable option in select patients. J Am Acad Dermatol.

[CR7] Council on Science and Research (2021) Position statement on superficial radiation therapy for basal cell carcinoma (BCC) and squamous cell carcinoma (SCC). AAD. https://server.aad.org/Forms/Policies/Uploads/PS/PS-Superficial%20Radiation%20Therapy.pdf? Accessed 12 Sept 2022

[CR8] Didona D, Paolino G, Bottoni U, Cantisani C (2018). Non melanoma skin cancer pathogenesis overview. Biomedicines.

[CR9] Global Burden of Disease Cancer Collaboration (2019). Global, regional and national cancer incidence, mortality, years of life lost, years lived with disability, and disability-adjusted life-years for 29 cancer groups, 1990 to 2017. JAMA Oncol.

[CR10] Gupta AK, Bharadwaj M, Mehrotra R (2016). Skin cancer concerns in people of color: risk factors and prevention. Asian Pac J Cancer Prev.

[CR11] Hamouzadeh P, Darkhor S, Aboie P, Zare M, Gray S (2017). Safety and effectiveness of superficial radiation therapy in the treatment of skin diseases: a systematic review. Heal Technol Assess Action.

[CR12] Iriyama S, Matsunaga Y, Takahashi K, Matsuzaki K, Kumagai N, Amano S (2011). Activation of heparanase by ultraviolet B irradiation leads to functional loss of basement membrane at the dermal-epidermal junction in human skin. Arch Dermatol Res.

[CR13] Kim GK, Del Rosso JQ, Bellew S (2009). Skin cancer in Asians part 1: nonmelanoma skin cancer. J Clin Aesthet Dermatol.

[CR14] Lavker RM, Zheng PS, Dong G (1989). Morphology of aged skin. Clin Geriatr Med.

[CR15] Leiter U, Eigentler T, Garbe C (2014). Epidemiology of skin cancer. Adv Exp Med Biol.

[CR16] Mansouri B, Housewright C (2017). The treatment of actinic keratoses—the rule rather than the exception. J Am Acad Dermatol.

[CR17] Meyer T (2009). Molecular events in skin cancer. Cancer Treat Res.

[CR18] Mohan SV, Chang AL (2014). Advanced basal cell carcinoma: epidemiology and therapeutic innovations. Current Dermatol Rep.

[CR19] Nestor MS, Berman B, Goldberg D (2019). Consensus guidelines on the use of superficial radiation therapy for treating nonmelanoma skin cancers and keloids. J Clin Aesthet Dermatol.

[CR20] Nikolaou V, Stratigos AJ (2014). Emerging trends in the epidemiology of melanoma. Br J Dermatol.

[CR21] Rogers HW, Weinstock MA, Feldman SR, Coldiron BM (2015). Incidence estimate of nonmelanoma skin cancer (keratinocyte carcinomas) in the US population, 2012. JAMA Dermatol.

[CR22] Samarasinghe V, Madan V (2012). Nonmelanoma skin cancer. J Cutan Aesthet Surg.

[CR23] The Lewin Group, Inc (2005) The burden of skin diseases 2005. Prepared for the Society for Investigative Dermatology, Cleveland, OH, and the American Academy of Dermatology Assn., Washington, DC

[CR24] Tran A, Gohara M (2021). Community engagement matters: a call for greater advocacy in dermatology. Int J Women's Dermatol.

[CR25] Tran A, Desai S, Robinson DM (2023). From ancient egypt to the dermatologic office: an overview of skin substitutes and modern day application. Health Sci Rep.

[CR26] Vaidya T, Zubritsky L, Alikhan A, Housholder A (2018). Socioeconomic and geographic barriers to dermatology care in urban and rural U.S. populations. J Am Acad Dermatol.

[CR27] Yu L, Moloney M, Beers R, Serure D (2021). Enhancing cosmesis while achieving high cure rates for early stage non-melanoma skin cancer in the outpatient dermatology clinic using a novel non-invasive modality. Clin Derm: Res Ther.

[CR28] Yu L, Oh C, Shea CR (2021). The treatment of non-melanoma skin cancer with image-guided superficial radiation therapy: an analysis of 2917 invasive and in situ keratinocytic carcinoma lesions. Oncol Ther.

[CR29] Yu L, Moloney M, Tran A, Zheng S, Rogers J (2022). Local control comparison of early-stage non-melanoma skin cancer (NMSC) treated by superficial radiotherapy (SRT) and external beam radiotherapy (XRT) with and without dermal image guidance: a meta-analysis. Discov Oncol.

